# The influence of social feedback on reward learning in the Iowa gambling task

**DOI:** 10.3389/fpsyg.2024.1292808

**Published:** 2024-05-02

**Authors:** Ming Peng, Qiaochu Duan, Xiaoying Yang, Rui Tang, Lei Zhang, Hanshu Zhang, Xu Li

**Affiliations:** ^1^Key Laboratory of Adolescent Cyberpsychology and Behavior (CCNU), Ministry of Education, Wuhan, China; ^2^School of Psychology, Central China Normal University, Wuhan, China; ^3^Key Laboratory of Human Development and Mental Health of Hubei Province, Wuhan, China; ^4^Centre for Human Brain Health, School of Psychology, University of Birmingham, Birmingham, United Kingdom; ^5^Institute for Mental Health, School of Psychology, University of Birmingham, Birmingham, United Kingdom

**Keywords:** social feedback, reward learning, Iowa gambling task, computational model, feedback type, identity

## Abstract

Learning, an important activity for both human and animals, has long been a focal point of research. During the learning process, subjects assimilate not only their own information but also information from others, a phenomenon known as social learning. While numerous studies have explored the impact of social feedback as a reward/punishment during learning, few studies have investigated whether social feedback facilitates or inhibits the learning of environmental rewards/punishments. This study aims to test the effects of social feedback on economic feedback and its cognitive processes by using the Iowa Gambling Task (IGT). One hundred ninety-two participants were recruited and categorized into one non-social feedback group and four social feedback groups. Participants in the social feedback groups were informed that after the outcome of each choice, they would also receive feedback from an online peer. This peer was a fictitious entity, with variations in identity (novice or expert) and feedback type (random or effective). The Outcome-Representation Learning model (ORL model) was used to quantify the cognitive components of learning. Behavioral results showed that both the identity of the peer and the type of feedback provided significantly influenced the deck selection, with effective social feedback increasing the ratio of chosen good decks. Results in the ORL model showed that the four social feedback groups exhibited lower learning rates for gain and loss compared to the nonsocial feedback group, which suggested, in the social feedback groups, the impact of the recent outcome on the update of value decreased. Parameters such as forgetfulness, win frequency, and deck perseverance in the expert-effective feedback group were significantly higher than those in the non-social feedback and expert-random feedback groups. These findings suggest that individuals proactively evaluate feedback providers and selectively adopt effective feedback to enhance learning.

## 1 Introduction

Learning is a central topic in psychological research and questions about learning have been addressed in virtually all areas of psychology (De Houwer et al., 2013). Social learning is broadly defined as learning from or through interaction with other individuals. This form of learning is often adaptive because it allows learning about the world while minimizing exposure to predation and other threats and offers access to others’ innovations (Olsson et al., 2020). People, even young children, draw rich inferences from the evidence provided by others and generate informative evidence that helps them learn (Gweon, 2021).

Social information can be gleaned either by observing others’ behavior (Charpentier et al., 2020; Zhang et al., 2020; Zhao et al., 2023) or by following explicit advice or social feedback (Harris, 2012; Colombo et al., 2014; Van der Borght et al., 2016; Hertz et al., 2021; Zonca et al., 2021; Schindler et al., 2022). Processing social feedback is essential for social learning, imitation, and adaptation; thus, it plays a crucial role in daily life (Vélez and Gweon, 2021; Zhang et al., 2022). Dozens of laboratory and field studies have shown that humans effectively shape others’ behavior through the use of selective rewards and punishments (Ho et al., 2017, 2019).

Compared with other social information, social feedback not only provides information about the world, but also provides a positive feeling (Ho et al., 2019). For example, teacher feedback can both improve achievement and foster pride. Therefore, there are at least two types of social feedback: social feedback itself as a reward/punishment, and social feedback that facilitates the function of an environmental or physical reward/punishment (usually a monetary incentive). For example, social feedback influences the processing of gain or loss in economic decisions (Namba, 2021). The following questions are worth investigating: does the acquisition of knowledge through social rewards and punishments differ from that of feedback derived from conventional environmental or physical rewards and punishments? How do the two distinct forms of feedback interact with one another? Although some evidence suggests that shared neural regions are involved in processing social and physical feedback (Izuma et al., 2008; Lin et al., 2012), the precise nature of their interactions remains largely unexplored. This study aims to address this knowledge gap.

The Iowa Gambling Task (IGT) is a reward-learning task relying on monetary feedback (Bechara et al., 1994). With the IGT, participants are required to choose four decks that will elicit feedback in the form of either a reward or punishment, and aim to obtain as great a reward as possible. Two of the decks have smaller immediate rewards, but result in greater net gains (classified as good decks), and two decks are associated with larger immediate rewards, but result in greater net losses (classified as bad decks; Bechara et al., 1994). Normally, participants in the IGT adopt an explore-exploit strategy in which they first explore different decks and then exploit the most profitable one when they find the best deck (Bechara et al., 1994; Must et al., 2006; Agay et al., 2010; Namba, 2021).

Many studies have focused on how different people react to environmental feedback in the IGT (Cauffman et al., 2010; Mukherjee and Kable, 2014; Hayes and Wedell, 2020b; Garon and English, 2021; Serrano et al., 2022). However, few recent studies have focused on the influence of social feedback in the task (Case and Olino, 2020). One study examined learning patterns in response to both monetary and social incentives using modified versions of the IGT in a sample of 191 undergraduate students. The social feedback consisted of facial images displaying positive and negative emotions. The results showed that participants demonstrated learning in both the monetary and social tasks, as shown by decreases in play on bad decks across the task. Additionally, they found that overall task performance on monetary and social tasks was associated with fun-seeking, and that performance on the social task was also associated with depressive symptoms (Case and Olino, 2020).

As mentioned before, social feedback can be used as a reward/punishment, or to facilitate the function of an environmental reward/punishment. Previous studies using the IGT have dealt with the former case (e.g., Lin et al., 2012; Thompson and Westwater, 2017; Case and Olino, 2020); however, to the best of our knowledge, only one study has investigated the latter case (Namba, 2021). That study investigated whether learning can be promoted by adding feedback in the form of facial expressions to the normal monetary feedback provided in the IGT. To ascertain the effect of facial-expression feedback, the researchers added a control condition that included feedback in the form of symbols (○ and ×). ○ has conventionally been used as feedback for positive or correct evaluations, while × has been used as feedback for negative or incorrect evaluations. These two conditions were similar in that both provided information and monetary feedback. The results revealed that the learning rate for facial expression feedback was slower in the middle of the task period than that for symbolic feedback (Namba, 2021). Although this study demonstrated that social feedback affects reward learning, the underlying mechanism remains unknown.

Researchers have studied the conditions under which individuals rely on information from social sources to inform their behavior, which is known as Social Learning Strategies (SLS) (Laland, 2004). These strategies, referred to as “transmission biases” or “heuristics” are thought to lead individuals to imitate certain behaviors (known as “what” strategies), performed by certain individuals (known as “who” strategies), in certain contexts (known as “when” strategies) (Kendal et al., 2018). However, they are not used indiscriminately. Through theoretical modeling and empirical evidence, it has been suggested that humans and non-human animals employ strategies such as copying when uncertain, copying the majority, and copying authoritative individuals, as the use of social information does not guarantee success (Heyes, 2012; Olsson et al., 2020). The objective of this study was to examine two distinct aspects of feedback, namely the effectiveness of feedback on “what” strategies and the role of the feedback provider’s identity in “who” strategies according to SLS.

Learning from social feedback is an important form of social learning, however, it is still unclear how social feedback affects the internal cognitive process of environmental reward/punishment learning. Using a computational model, we can analyze the decision-making process into its components. Multiple computational models have been proposed, three of which were proposed by Ahn and colleagues, including the Prospect Valence Learning model with Delta rule (PVL-Delta) (Ahn et al., 2008), Value-Plus-Perseverance model (VPP) (Ahn et al., 2014), and Outcome-Representation Learning model (ORL) (Haines et al., 2018). Their latest model, ORL, contains five free parameters, reward learning (A_rew_), punishment learning (A_pun_), forgetfulness (K), win frequency (β_F_), and deck perseverance (β_P_). A_rew_ (0 < A_rew_ < 1) and A_pun_ (0 < A_pun_ < 1) are learning rates used to update expectations after reward (i.e., positive) and punishment (i.e., negative) outcomes, respectively. When the learning rate is high, the most recent outcomes matter for the value update, whereas when the learning rate is low, the impact of the value of the most recent outcomes on the value update decreases (Rescorla and Wagner, 1972; Zhang et al., 2020). K is a decay parameter that controls how quickly decision-makers forget their past deck choices, where lower values imply longer lasting memories of past choices. Values for β_F_ (−∞ < β_F_ < +∞) less than or greater than 0 indicate that people prefer decks with a low or high win frequency, and values for β_P_ (−∞ < β_P_ < +∞) less or greater than 0 indicate that people prefer to switch or stay with recently chosen decks. ORL outperformed the previous two learning reinforcement models (PVL-Delta and VPP) in terms of prediction accuracy and parameter recovery (Haines et al., 2018).

We integrate the identity and behavioral characteristics of feedback providers to examine how different characteristics affect an individual’s learning. Two different learning performances were used. The first pertains to the chosen rate of good decks, serving as an indicator of behavioral performance. We hypothesized that better task performance would be observed when the social feedback was more effective than random or no social feedback. The second is the learning rate (A_rew_ and A_pun_), which was an index of internal cognitive processes, analyzed through a computational model. In examining the relationship between learning rate and performance, findings have been inconsistent (Cutler et al., 2021; Westhoff et al., 2021). Notably, regarding whether a higher learning rate leads to better performance, we propose that in situations characterized by environmental stability and adequate instructional guidance, a lower learning rate is anticipated. Therefore, we hypothesized that a lower learning rate would be observed when feedback is effective and when the feedback provider is an expert.

## 2 Methods

### 2.1 Participants

A prior power analysis was conducted using G*Power v.3.1 (Faul et al., 2007) to determine the sample size for the nonsocial and social feedback settings. For the nonsocial setting, 24 participants were required with an alpha of 0.05, power (1 – β) of 0.80, and a medium effect size of 0.25 for the within-group effect. For the social setting, 128 participants were required with an alpha of 0.05, power of 0.80, and a medium effect size of 0.25 for the within-between interaction effect. In total, 39 participants (25 females, mean ± SE = 20.6 ± 2.6) were recruited for the nonsocial setting and 153 participants (109 females, mean ± SE = 20.1 ± 2.1) were recruited for the social setting from a university located in Wuhan, Hubei province.

All participants were in good physical and mental health and were informed of the experiment procedure, rewards, and risks. Monetary rewards were dispensed after the experiment based on participants’ performance, with a range of 8–10 Chinese yuan given as a participation fee.

### 2.2 Iowa gambling task

Participants in the modified version of the Iowa Gambling Task (IGT) were presented with four decks labeled D, F, J, and K, which corresponded to four specific decks of cards randomly assigned (referred to as A, B, C, and D). Each deck of cards had two properties: gain and loss. The good decks (C and D) had an expected value of 25 yuan, while the bad decks (A and B) had an expected value of −25 yuan. Additionally, the decks differed in loss frequency: good deck C and bad deck A had frequent mixed outcomes (5 losses out of every 10 cards), while the other decks (B and D) had infrequent mixed outcomes (1 loss out of every 10 cards). The starting outcome was 0. The detailed payoff was shown in Table 1.

**Table 1:** The schedule of gain and loss in the four decks of the card task used in the task.

Deck	Outcome	Payoff in per 10 trials										Net
A	Gain	8	9	10	11	12	8	9	10	11	12	−2.5
	Loss	0	0	0	0	0	−15	−20	−25	−30	−35	
B	Gain	8	9	10	11	12	8	9	10	11	12	−2.5
	Loss	0	0	0	0	0	0	0	0	0	−125	
C	Gain	3	4	5	6	7	3	4	5	6	7	2.5
	Loss	0	0	0	0	0	−1	−3	−5	−7	−9	
D	Gain	3	4	5	6	7	3	4	5	6	7	2.5
	Loss	0	0	0	0	0	0	0	0	0	−25	

Participants were randomized in terms of deck position, but the deck labels were always displayed in order from left to right, corresponding to the keyboard keys’ labels. Each trial, participants pressed the corresponding key.

In the non-social feedback setting, participants completed the task alone. For the social feedback setting, participants were divided into four groups and given feedback from computer-mocked partners. The partners varied in terms of feedback type and identity: those with effective feedback gave supportive feedbacks for 80 percent of good deck choices and disapproving feedbacks for 80 percent of bad deck choices, while those with random feedback gave supportive feedbacks for 80 percent of all choices and disapproving feedbacks for 20 percent of all choices. The identities of the partners were set as novices (who knew nothing about the task) and experts (who had already learnt how to find better decks). The identity of the partner was introduced to the participants before the task began.

### 2.3 Procedure

Participants were tested individually in both a non-social and social feedback setting. In the social feedback setting, they were informed that they were collaborating with an anonymous partner online, who shared the same experiment screen content. Participants were given instructions on the computer screen. The experimenter highlighted the importance of winning as much money as possible, with their remuneration being contingent on the final outcome. For the social feedback group, the partner was either ignorant of the task (in the novice group) or had been provided instructions on how to find better decks (in the expert group). In actuality, the partner was simulated by a computer program and the social feedbacks were generated by a program.

**Figure 1:**
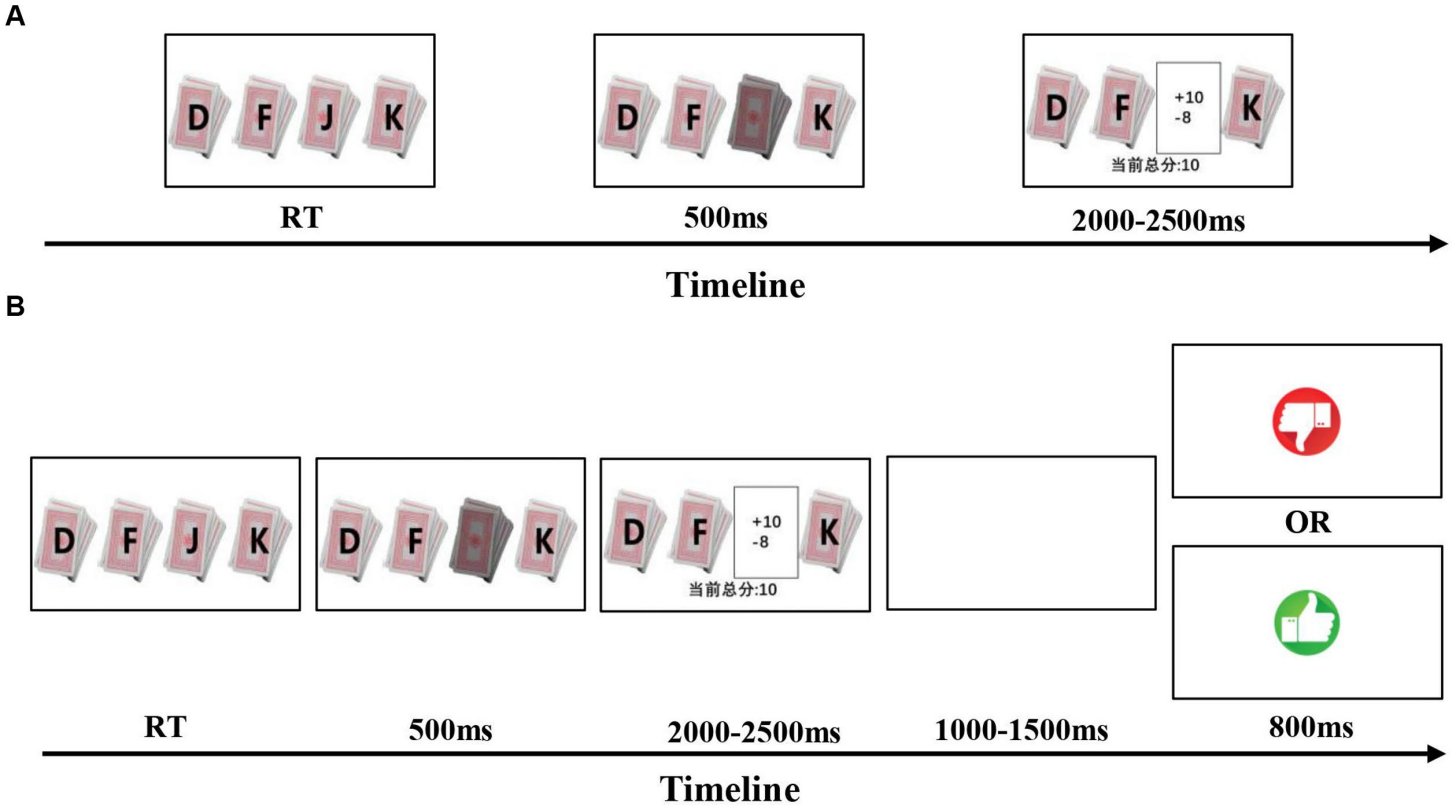
Experiments design: **(A)** non-social feedback condition: participants made choice from four decks and received monetary feedbacks. **(B)** Social feedback condition: participants chose and got monetary feedbacks as non-social condition, then they received their partners’ feedback shown as picture of thumb up or down while the partner was played by computer.

The experiment lasted approximately 20–30 min and began with four decks labeled “D,” “F,” “J,” and “K.” After participants selected a deck, the choice monetary feedback was displayed for 2–2.5 s. In the social feedback setting, participants waited 1–1.5 s after receiving the choice monetary feedback, followed by the partner’s feedback (a finger up or down picture) for 0.8 s. The task was completed after participants finished 120 trials or two decks were all chosen. The flow chart is Figure 1.

### 2.4 Behavioral data process

A repeated-measure ANOVA of 4 blocks (1–30 trials, 31–60 trials, 61–90 trials, 91–120 trials) was used to analyze the chosen rate of good decks and the group switch rate in the non-social feedback setting. Similarly, a mixed-measure ANOVA of 4 blocks (1–30 trials, 31–60 trials, 61–90 trials, 91–120 trials) × 2 feedback types (random, effective) × 2 identities (novice, expert), as identity and feedback type were between-subjects, was used to analyze the chosen rate of good decks and the group switch rate in the social feedback setting. This was done to investigate the participants’ behavior changing tendencies. The higher chosen rate of good decks and the higher switch rate indicated better performance in seizing the pivot of the task and higher exploratory tendencies, respectively. We also did a one-way ANOVA of group (non-social group, novice-random group, novice-effective group, expert-random group, expert-effective group) on the slope of chosen rate to get the rate at which different groups of individuals learn.

The behavioral results were fitted into three models: the Prospect Valence Learning model with the delta model, the Value-Plus-Perseverance model, and the Outcome-Representation model. The detailed information of these three models was presented in the Supplementary material. The analyses were conducted using the hbayesDM package (Ahn et al., 2017) in R (4.1.3) with an iteration of 20,000. This package utilizes hierarchical Bayesian modeling, which is more stable than traditional fitting methods such as maximum likelihood estimation, and computes both group and subject level parameters. The model parameters’ distributions and the leave one out information criterion (LOOIC) were obtained. The lower the LOOIC, the better the model is. To assess the effect of social feedback on learning, the mean of parameters from each social and nonsocial feedback group were compared. The results were the posterior distribution of mean differences of each parameter that came from four social feedback groups’ parameters distribution minus that of the nonsocial group. In the social feedback setting, the model parameters were each analyzed using a between-subjects ANOVA of 2 (feedback type: random, effective) × 2 (identity: novice, expert). IBM SPSS Statistics 27, MATLAB R2020b and R 4.1.3 were used for data analysis and model calculation.

## 3 Results

### 3.1 Behavior results

**Figure 2:**
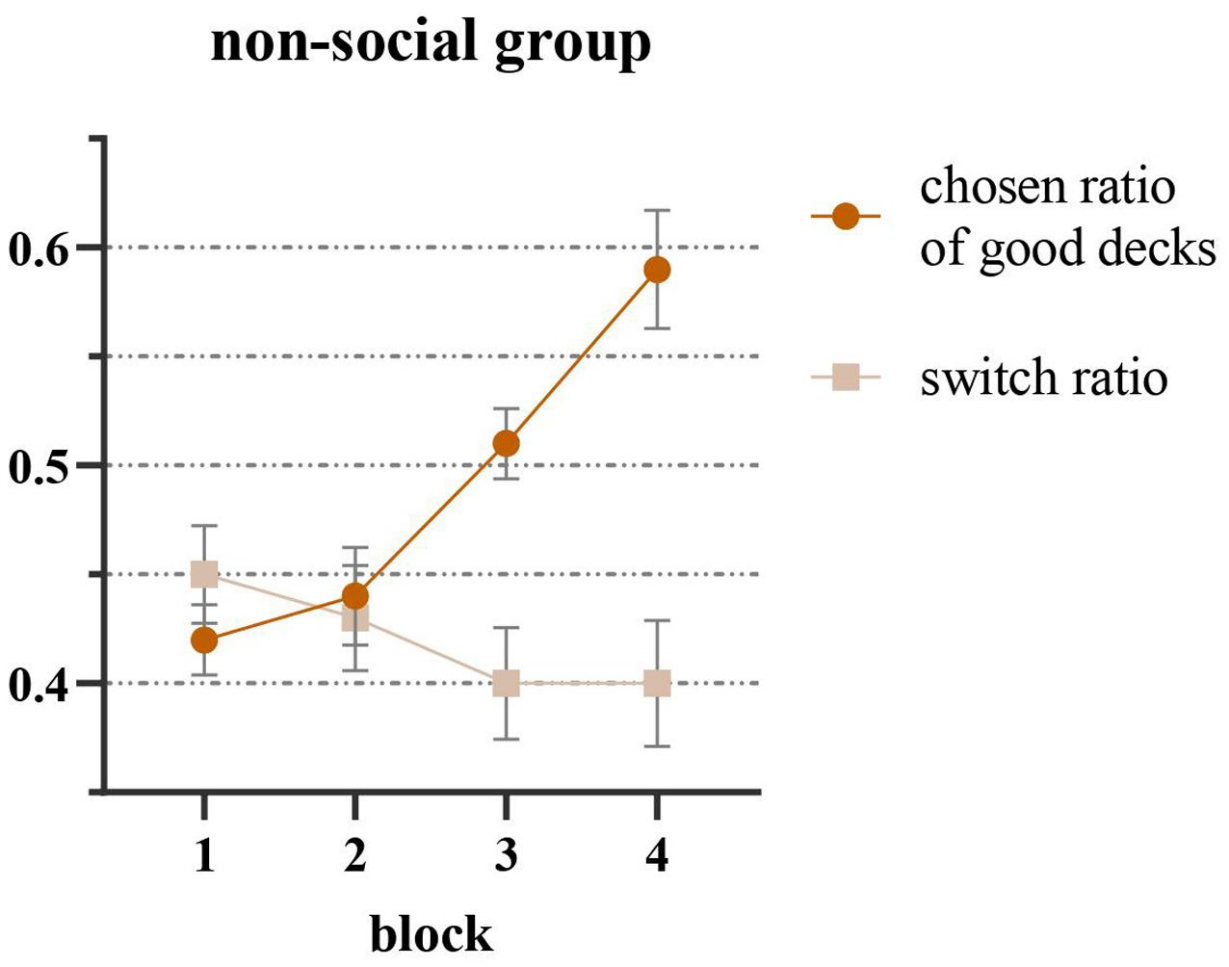
Results of non-social feedback group that the chosen ratio of good decks was the trials number each block divided by the times of choosing C/D and the switch ratio was the trials number each block divided by the times of changes between good/bad decks. The chosen ratio of good decks increased by blocks significantly and the switch ratio decreased by blocks.

An analysis of the effect of decision-making blocks on the chosen rate of good decks (C, D) and the group switch rate in the non-social feedback group revealed a significant difference in the former (*F* (3,111) = 15.31, *p* < 0.001, η*_p_*^2^ = 0.29). Bonferroni’s multiple comparisons indicated that the chosen rate of good decks in block 3 was significantly higher than that in block 1 (*p* < 0.001), and block 4 was significantly higher than block 1 (*p* < 0.001), block 2 (*p* = 0.001) and block 3 (*p* = 0.019), suggesting that participants demonstrated a learning effect on the decks’ properties and an increase in the chosen rate of good decks as the decision-making process progressed (Figure 2). No significant difference in the group switch rate among blocks was observed (*F* (3,111) = 1.29, *p* = 0.23).

**Figure 3:**
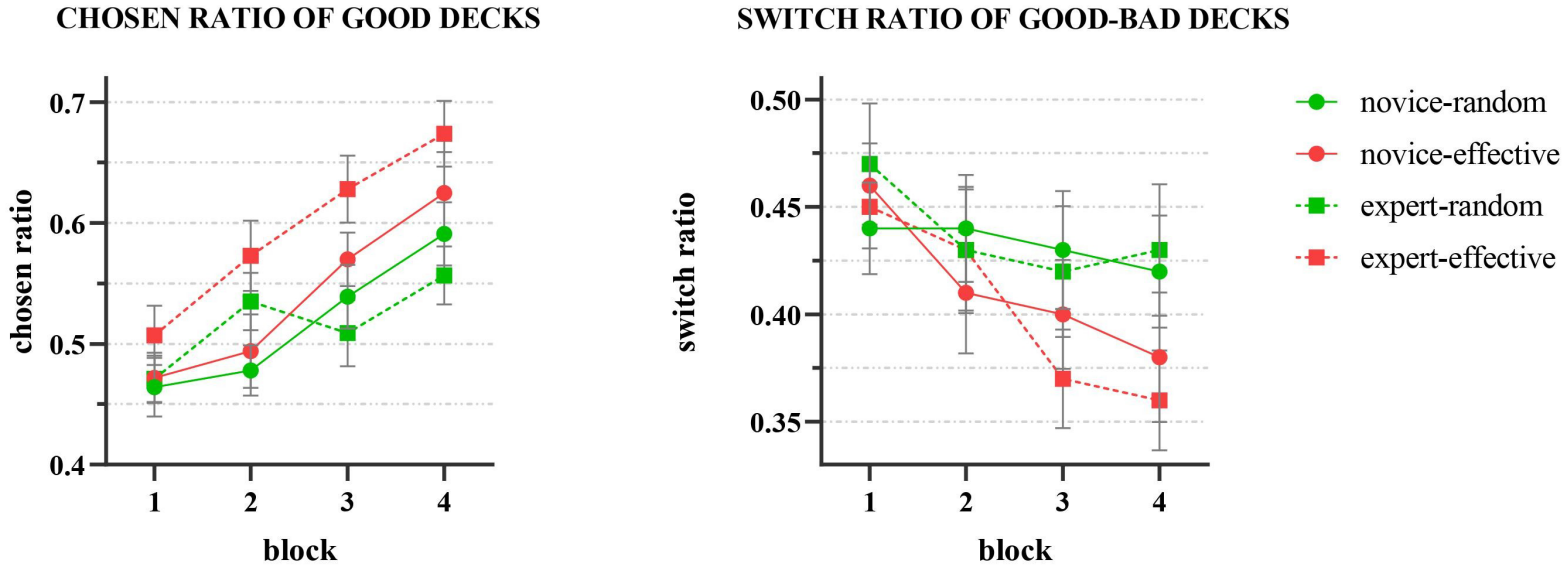
Results of social feedback groups that the chosen ratio of good decks increased by blocks significantly and the switch ratio decreased by blocks.

In the social feedback group (Figure 3), a significant main effect of decision-making blocks on the chosen rate of good decks (C, D) was observed (*F* (3,447) = 20.66, *p* < 0.001, η*_p_*^2^ = 0.12). Bonferroni’s multiple comparisons revealed that the chosen rate of good decks in block 4 was significantly higher than that in block 1 (*p* < 0.001), block 2 (*p* < 0.001), and block 3 (*p* = 0.006), indicating that participants demonstrated a learning effect on the decks’ properties and an increase in the chosen rate of good decks as the decision-making process progressed. Additionally, a significant main effect of partner’s identity (*F* (1, 149) = 4.18, *p* = 0.043, η*_p_*^2^ = 0.03) and feedback type (*F* (1, 149) = 11.73, *p* < 0.001, η*_p_*^2^ = 0.07) was observed, with the chosen rate of good decks being significantly higher when the partner was an expert than a novice, and the chosen rate of good decks in effective feedback being significantly higher than that in random feedback. A marginal significant interaction effect was observed between feedback type and partner identity (*F* (1, 149) = 3.58, *p* = 0.06) that the chosen rate of good decks was significantly higher in valid group than in random group when the partner was an expert *p* < 0.001. When the partner was a novice, there was no significant difference between two feedback groups. The result on one-way ANOVA of group on the slope of chosen rate showed no significant difference on the slope, *F* (4, 191) = 0.605, *p* = 0.659.

Our results indicated that decision-making blocks had a significant effect on group switch rate (*F* (3,447) = 7.08, *p* < 0.001, η*_p_*^2^ = 0.05). Bonferroni’s multiple comparisons showed that the group switch rate in block 4 was significantly lower than that in blocks 1 and 2 (*p* = 0.015). This suggests that, as decision-making progresses, participants exhibit a decrease in group switch rate. Neither partner’s identity (*F* (1, 149) = 0.07, *p* = 0.80) nor feedback type (*F* (1, 149) = 2.13, *p* = 0.15) had a significant main effect, and there was no significant interaction between the two (*F* (1, 149) = 0.169*, p* = 0.682).

### 3.2 Model comparison

An analysis of the model performance between the PVL-delta model, VPP model and ORL model with the data of non-social and social feedback groups, as shown in Table 2, demonstrates that the ORL model is the best fit.

**Table 2:** Results of model comparison.

	LOOIC		
	PVL-delta	VPP	ORL
Non-social	12215.56	11281.95	11251.08
Novice-random	13172.05	12406.50	12285.86
Novice-effective	11614.71	10786.92	10729.26
Expert-random	11850.38	10962.58	10922.58
Expert-effective	11205.61	10740.78	10691.33

### 3.3 Parameter analysis

**Figure 4:**
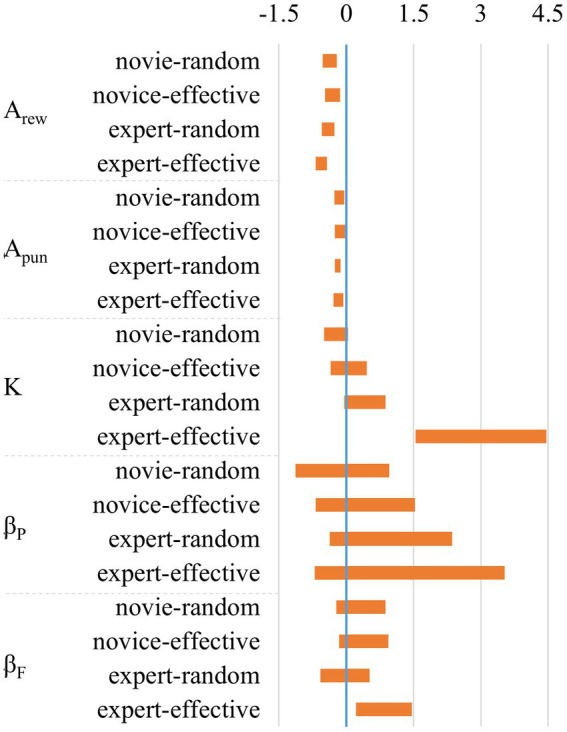
HDI differences of parameters between non-social and social feedback conditions.

The comparison of the posterior distribution of mean differences between non-social feedback group and each social feedback group showed that the A_rew_ and A_pun_ of the non-social feedback group were significantly higher than those of the social feedback group, as Figure 4 and Table 3 show, with the HDI differences of A_rew_ and A_pun_ between the two groups being distributed away from zero. Additionally, the K and β_F_ of the expert-effective group were significantly higher than those of the non-social feedback group, with the HDI differences of K and β_F_ between the two groups also being distributed away from zero.

**Table 3:** Differences of the posterior distribution of parameters mean between non-social and social condition (HDI).

	Difference with non-social group				
	A_rew_	A_pun_	K	β_P_	β_F_
Novice-random	[−0.523, −0.210]	[−0.267, −0.047]	[−0.492,0.032]	[−1.131,0.957]	[−0.220, 0.870]
Novice-effective	[−0.469, −0.139]	[−0.256, −0.034]	[−0.351, 0.458]	[−0.679, 1.529]	[−0.157, 0.934]
Expert-random	[−0.549, −0.261]	[−0.321, −0.133]	[−0.045, 0.877]	[−0.371, 2.360]	[−0.575, 0.518]
Expert-effective	[−0.549, −0.261]	[−0.285, −0.071]	[1.545,4.457]	[−0.371, 2.360]	[0.213,1.456]

**Figure 5:**
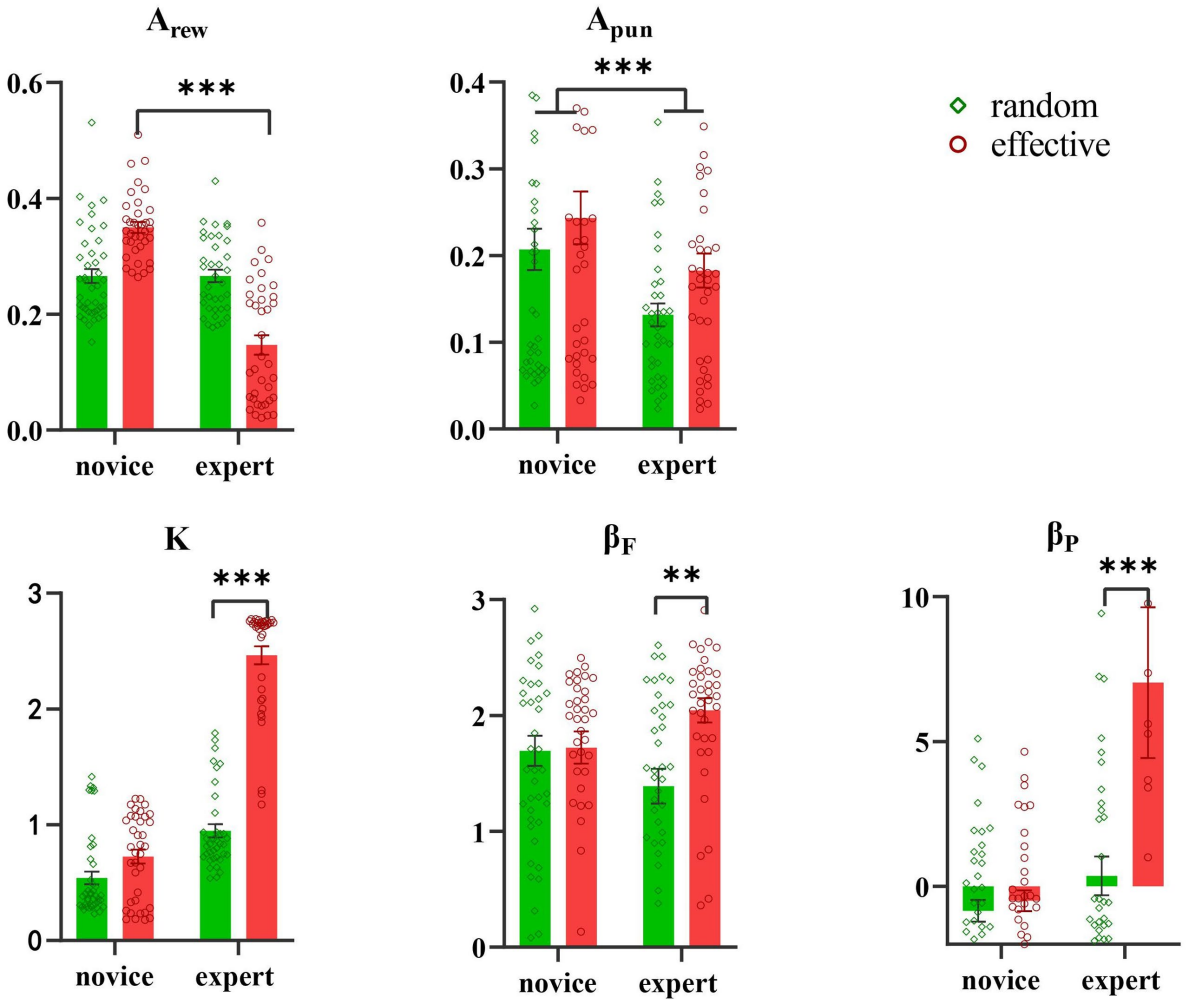
Results of ANOVA in social feedback groups.

A between-subjects ANOVA analysis was conducted for the social feedback condition, with the results shown in Figure 5 and Table 4. The results revealed a significant main effect of partner’s identity (*F* (1, 153) = 65.67, *p* < 0.001, η*_p_*^2^ = 0.30) on A_rew_, but no significant main effect of feedback type (*F* (1, 153) = 1.97, *p* = 0.16). Additionally, a significant interaction effect of partner’s identity × feedback type was observed (*F* (1, 153) = 65.89, *p* < 0.001, η*_p_*^2^ = 0.31). Further simple effect analysis indicated that A_rew_ of novices was significantly higher than that of experts in effective feedback (*F* (1, 152) = 127.65, *p* < 0.001, η*_p_*^2^ = 0.46), but there was no significant difference between novice and expert when in random feedback (*F* (1, 152) = 0.00, *p* = 0.96).

**Table 4:** Values of parameters in social condition.

Identity	Type	A_rew_	A_pun_	K	β_F_	β_P_
Novice	Random	0.27 ± 0.08	0.21 ± 0.15	0.54 ± 0.36	1.69 ± 0.84	−0.84 ± 2.44
	Effective	0.35 ± 0.06	0.24 ± 0.19	0.73 ± 0.37	1.72 ± 0.85	−0.49 ± 2.20
Expert	Random	0.27 ± 0.07	0.13 ± 0.08	0.95 ± 0.35	1.39 ± 0.92	0.36 ± 4.06
	Effective	0.15 ± 0.10	0.18 ± 0.12	2.45 ± 0.47	2.04 ± 0.64	7.03 ± 11.95

Results revealed a significant main effect of partner’s identity on A_pun_ parameters (*F* (1, 153) = 8.87, *p* = 0.003, η*_p_*^2^ = 0.06), with novice A_pun_ being higher than that of expert. The main effect of feedback types was not significant (*F* (1, 153) = 3.65, *p* = 0.06), and no interaction effect between feedback type and partner’s identity was observed (*F* (1, 153) = 0.11, *p* = 0.74).

Analysis of K parameters revealed a significant main effect of partner’s identity (*F* (1, 153) = 292.04, *p* < 0.001, η*_p_*^2^ = 0.66) and feedback type (*F* (1, 153) = 183.44, *p* < 0.001, η*_p_*^2^ = 0.55), in addition to a significant interaction effect of partner’s identity × feedback type (*F* (1, 153) = 112.45, *p* < 0.001, η*_p_*^2^ = 0.43). Further examination indicated that *K* values were significantly higher when the partner was an expert and the feedback was effective, compared to when it was random (*F* (1, 152) = 96.21, *p* < 0.001, η*_p_*^2^ = 0.39). However, there was no significant difference between novice and expert when both effective and random feedback was used (*F* (1, 152) = 2.80, *p* = 0.10).

Results revealed a significant main effect of feedback type (*F* (1, 153) = 6.66, *p* = 0.011, η*_p_*^2^ = 0.04) on β_F_. However, the main effect of partner’s identity was not significant (*F* (1, 153) = 0.00, *p* = 0.95). Additionally, a significant interaction effect of partner’s identity × feedback type was observed (*F* (1, 153) = 5.56, *p* = 0.020, η*_p_*^2^ = 0.04). Subsequent simple effect analysis revealed that the β_F_ of effective feedback was significantly higher than that of random feedback when the partner was an expert (*F* (1, 152) = 11.91, *p* < 0.001, η*_p_*^2^ = 0.07), but there was no significant difference between random feedback and effective feedback when the partner was a novice (*F* (1, 152) = 0.03, *p* = 0.87).

A significant main effect of partner’s identity (*F* (1, 153) = 10.81, *p* < 0.001, η*_p_*^2^ = 0.07) and feedback type (*F* (1, 153) = 6.99, *p* = 0.009, η*_p_*^2^ = 0.05) was observed for β_P_ parameters. Additionally, a significant interaction effect of partner’s identity and feedback type was also seen (*F* (1, 153) = 5.67, *p* = 0.019, η*_p_*^2^ = 0.04). Subsequent simple effect analysis revealed that the β_P_ for effective feedback was higher than that of random feedback when the partner was an expert (*F* (1, 152) = 11.49, *p* < 0.001, η*_p_*^2^ = 0.07), however, there was no significant difference between random feedback and effective feedback when the partner was a novice (*F* (1, 152) = 0.10, *p* = 0.75).

## 4 Discussion

This study employed the Iowa Gambling Task (IGT) to examine the impact of social feedback on economic feedback. We utilized the IGT with and without social feedback and evaluated three computational models, finding that the Outcome-Representation Learning (ORL) model displayed the most successful performance in all five conditions. Subsequently, we explored the effects of the identity of the feedback provider and the type of feedback on learning behavior and cognitive process. The results indicated that the chosen rate of good decks was affected by the identity and type of feedback, respectively. Moreover, the parameters in the ORL model were differently impacted by identity, type, and the interaction between them.

Consistent with previous studies on the IGT, participants in a non-social feedback setting showed a significant difference in the ratio of chosen good decks across the blocks. As the experiment progressed, a gradual learning of the characteristics of the cards was observed, with an increased preference for the good decks (Bechara et al., 1994; Cassotti et al., 2014; Zhang et al., 2022). However, contrary to Bechara et al.’s (1994) hypothesis that the switch between options would become less frequent as the experiment went on, the rate of switching between the good and bad decks, as well as between decks within each category, showed no significant change by the end of the experiment, suggesting that the persistence of choice remained constant. This result is not unusual in previous studies, Steingroever et al. (2012) reviewed studies that used the original or modified versions of the IGT and found that participants did not demonstrate a systematic decrease in the number of switches across trials.

In the social feedback setting of the IGT task, feedback providers were divided into novices and experts and feedback type was divided into random and effective feedback, in order to explore the influence of feedback type and provider identity on learning. Results showed that participants gradually favored the good card decks, indicating that they had learned the characteristics of the card decks. Furthermore, the feedback provider’s identity and type had an effect on the selection ratio of the good decks, with the expert feedback group and the effective feedback group selecting the good decks significantly more than the novice feedback group and the random feedback group, respectively. Additionally, a marginal significant interaction effect was observed between identity and type, indicating that participants in the expert group were more likely to select good decks in the effective feedback group than in the random feedback group. This indicated that the subjects pay more attention to the feedback of experts, but they do not blindly follow the feedback of experts. Only effective feedback of experts can significantly increase the learning of the subjects. If feedback providers are novice, the subjects will not pay much attention to their feedback, so whether their feedback is effective or not, the difference in deck selection is not significant. It’s worth noting that the slope of chosen rate on good decks did not differ across five groups. This suggested that, based on the current task (IGT) and two types of social feedback (approving or disapproving), while effective social feedback can lead to an overall improvement in performance, it does not accelerate the learning process. However, it remains possible that social feedback in a different learning task or under varied social feedback conditions could accelerate learning.

The switch rate between the good and bad decks in the social feedback setting was significantly impacted by the decision block. As the experiment progressed, the switch rate between the two decks decreased significantly. Participants, at first, tended to explore to alleviate their uncertainties in beliefs, as demonstrated by their higher rate of choice switching in block 1 and block 2 compared to block 4. When they had gathered enough information, they then proceeded to exploit it (Hofmans and van den Bos, 2022). There was no difference between the four groups, indicating that the behavioral choices of the four groups were gradually becoming stable.

The result of model comparison revealed that the ORL model was the best-performing model in five groups after fitting the data in the PVL-delta, VPP, and ORL models and comparing the results. Theoretically, according to the ORL model, participants in the IGT learned the valence of the options (A_rew_ and A_pun_), deliberated on the effect of the loss probability of the options (β_F_), and showed an inclination to persist with their prior decisions (β_P_). Meanwhile, individuals exhibited variance in their recollection of their deck selection (K).

A_rew_ and A_pun_, the computational model parameters, are reflective of the participants’ learning degree on the current outcome of gains and losses decks. The comparison of the posterior distribution of mean differences between non-social feedback group and each social feedback group showed that compared to the non-social feedback group, the four social feedback groups exhibited lower rates of gains and losses learning. Furthermore, the gains learning rate (A_rew_) of the participants in the non-social feedback group was significantly higher than that of the expert feedback group in the effective feedback group, and the losses learning rate (A_pun_) of the novice feedback group was significantly higher than that of the expert feedback group regardless of effective or random feedback. The evidence indicated that in the absence of effective feedback, individuals displayed an increased weight of value of recent outcomes on the value update.

Previous studies have yielded inconsistent results regarding the relationship between learning rate and task performance. Cutler et al. (2021) found that individuals with higher learning rates performed better in reinforcement learning tasks when conducted in young and elderly groups under different reward recipient conditions. Conversely, Westhoff et al. (2021) observed that the learning rate decreased with age and task performance improved in probabilistic reinforcement learning tasks among children and adolescents. These divergent findings can be attributed to the uncertainty of gains and losses in the experimental environment. A lower learning rate in a stable yet ambiguous environment allows for better comprehension of environmental information, while a higher learning rate in a changing environment helps capture large fluctuations in the value of options. The task in this study resembled a stable environment (Hayes and Wedell, 2020a,b), suggesting that the subjects’ low learning rate likely contributes to their enhanced performance. In addition, this study incorporated two types of feedback, economic feedback (gain or loss) and social feedback (approving or disapproving), however, only the learning of economic feedback is included in the model. It is also possible that effective social feedback could lead subjects to learn from both social feedback and economic feedback (Hofmans and van den Bos, 2022), potentially resulting in a decrease in the learning rate of economic feedback. However, this hypothesis requires further investigation.

In the task, the frequency of losses varies for each deck of cards, and the computational model parameter βF indicates how much the outcome frequency influences the participants’ evaluation of options (Haines et al., 2018). Parameter K reflects the influence of preceding trials (Haines et al., 2018). A higher K in the expert-effective group implies that participants are considering more recent options. The results of the study showed that the β_F_ and K parameters in the expert-effective feedback group were significantly higher than those in the non-social feedback and expert-random feedback groups, respectively. This suggests that participants were more likely to consider win frequency across trials and more recent options when provided with effective guidance (Haines et al., 2018). Additionally, the β_P_ parameter in the expert-effective group was significantly higher than that in the expert-random feedback group, indicating that participants had a greater degree of persistence in the process of option value formation when provided with effective feedback. As experts provided feedback, learners verified its effectiveness, leading to discrepancies between expert-effective feedback and expert-random feedback.

The results of this study indicated that there was no significant difference between the effective and random groups in the novice feedback group for the three parameters, β_F_, β_P_ and K. Vélez and Gweon (2021) postulates that in the process of social learning, individuals not only process the information itself, but also assess the agent providing the information. If the content or accuracy of the information aligns with the identity of the agent, the individual’s evaluation of the agent increases and the weight of the information provided is amplified. Conversely, if the agent is deemed to be a novice, the participant may deem the feedback to be less informative, thus reducing the weight of the information provided. This study demonstrated that participants formed expectations for the effectiveness of feedback based on the peer’s past knowledge and experience, and when the peer was a novice, the participants thought that their feedback might not be very informative. Even if the peer provided effective feedback, these three parameters were still little affected.

Results of the analysis of the ratio of chosen good decks and the three parameters of β_F_, β_P_ and K revealed an interaction effect of identity and type. It was observed that participants’ perception of the feedback providers (whether they were experts or not) had an influence on the extent to which they considered the opinion and evaluated the effectiveness of the feedback. If they found that the opinions of experts were ineffective, they would reduce the influence of social feedback. This can be explained by the ‘when’ strategy of SLS, which suggests that when participants lack sufficient information to make optimal decisions in the IGT, they tend to rely on information from others, especially in uncertain situations. Additionally, the ‘who’ strategy, which entails taking cues from individuals who are more knowledgeable or experienced with the task, may also play a significant role (Olsson et al., 2020).

This study has certain limitations that should be noted. Firstly, in this study, we used two kinds of feedback, economic feedback and social feedback, and subjects would consider both kinds of feedback to determine their behavior during IGT tasks. However, the ORL model did not incorporate social feedback. Future research could design various models that incorporate social feedback to reveal how people integrate them. Secondly, in our experiment, the ratio of positive and negative feedback is 80:20 in all social feedback groups. However, in the effective feedback group, the average rate of good decks was less than 60% in the whole task, so the positive feedback ratio of subjects in the effective feedback group was generally lower than 80%, which would cause the imbalanced frequencies of positive feedback between the random feedback group and the effective feedback group. Because social feedback could provide a positive feeling (Ho et al., 2019), a higher ratio of positive feedback in the random group may lead to stronger positive feelings among participants in that group than in the effective feedback group. Third, the feedback, whether in the random or effective group, is pseudo-social and constant throughout the experiment, potentially limiting its credibility. Further studies could use models to establish the behavior pattern of feedback that simulates real feedback, or explore the real two-person task scenario to update parameters within a one-person computational model. Fourthly, the sex ratio of the participants was unbalanced, with more women than men, thus it was not possible to investigate whether gender had an effect on learning differences. Lastly, this study investigated the impact of peer feedback in terms of behavioral performance and computational model parameters, without considering the influence of individual subjective feelings and individual differences. Future experiments should therefore include the measurement of subjective feelings such as subjective engagement and trust in peers, as well as individual characteristics.

## Data availability statement

The raw data supporting the conclusions of this article will be made available by the authors, without undue reservation.

## Ethics statement

The studies involving humans were approved by Central China Normal University, Ethic Committee, EC, Institutional Review Board. The studies were conducted in accordance with the local legislation and institutional requirements. The participants provided their written informed consent to participate in this study.

## Author contributions

MP: Conceptualization, Writing – review & editing, Methodology, Project administration, Resources, Supervision, Validation, Writing – original draft. QD: Formal analysis, Methodology, Software, Writing – original draft. XY: Methodology, Project administration, Writing – original draft. RT: Methodology, Project administration, Writing – original draft. LZ: Supervision, Writing – review & editing. HZ: Supervision, Writing – review & editing. XL: Writing – review & editing.
