# Supplementary material for: The influence of social feedback on reward learning in the Iowa gambling task
Source: Front Psychol. 2024 May 2;15:1292808. doi: 10.3389/fpsyg.2024.1292808 (PMC11098015; doi:10.3389/fpsyg.2024.1292808)
Supplement: Supplementary file 1 [file Table_1.DOCX]

## Supplement: computational models

### Prospect Valence Learning model with delta rule (PVL-delta)

The PVL-Delta model uses a prospect theory utility function to transform realized, objective monetary outcomes into subjective utilities:

$u\left( t \right)=\left\{ \begin{aligned} x\left( t \right)^{\alpha},x\left( t \right)>0 \\ -\lambda\left| x\left( t \right) \right|^{\alpha},x\left( t \right)\leq0 \end{aligned} \right.$

*t*: trial number; *u (t)*: subjective utility of the outcome; *x (t):* net outcome; α: parameter govern the shape of the utility function; λ: loss sensitivity;

The PVL-Delta model uses a simplified variant of the Rescorla–Wagner rule to update expected values of each deck:

$E_{j}\left( t+1 \right)=E_{j}\left( t \right)+A\cdot\left( u\left( t \right)-E_{\dot{j}}\left( t \right) \right)$

*E_j_ (t)* : the expected value of chosen deck *j* on trial *t*; *A*: learning rate;

Expected values are entered into a Softmax function to generate choice probabilities:

$P_{r}\left[ D\left( t \right)=j \right]=\frac{ⅇ^{\theta E_{j}\left( t \right)}}{\Sigma_{k=1}^{4}ⅇ^{\theta E_{k}\left( t \right)}}$

$\theta=3^{c}-1$

*D (t)*: the chosen deck on trial *t*; *c*: trial-independent choice consistency;

The PVL-Delta model contains four free parameters (*A*, α, *c*, λ).

### Value-Plus-Perseverance model (VPP)

The VPP model expands upon the PVL-Delta model by adding an additional term for choice perseverance:

$P_{j}\left( t+1 \right)=\left\{ \begin{aligned} K\cdot P_{j}\left( t \right)+\varepsilon_{P,}x(t)\geq0 \\ K\cdot P_{j}\left( t \right)+\varepsilon_{N,}x(t)<0 \end{aligned} \right.$

*P_j_ (t)*: the perseveration value for chosen deck *j* on trial *t*; *K*: decay parameter; ε*_P_*_,_ ε*_N_* : perseveration tendencies;

The VPP integrated the expected value (from the PVL-Delta model) and perseveration terms into a single value signal:

$V_{j}\left( t+1 \right)=w\cdot E_{j}\left( t+1 \right)+\left( 1-w \right)\cdot P_{j}\left( t+1 \right)$

*w*: weight parameter;

The VPP uses the same Softmax function as the PVL-Delta to generate choice probabilities, except that *E_j_ (t)* replaced with *V_j_ (t)*. The VPP contains eight free parameters (*A*, α, *c*, λ, ε*_P_*_,_ ε*_N,_ K, w*).

### Outcome-Representation Learning model (ORL)

The ORL assumes that the expected value and win frequency for each deck are tracked separately and the expected value of a deck is updated with separate learning rates for positive and negative outcomes:

$EV_{j}\left( t+1 \right)=\left\{ \begin{aligned} EV_{j}\left( t \right)+A_{rew}\cdot\left( x\left( t \right)-EV_{j}\left( t \right) \right),x\left( t \right)\geq0 \\ EV_{j}\left( t \right){+A}_{pun}\cdot\left( x\left( t \right)-EV_{j}\left( t \right) \right),x\left( t \right)<0 \end{aligned} \right.$

*EV_j_ (t)*: the expected value of chosen deck *j* on trial *t*; A_rew_, A_pun_: learning rates which are used to update expectations after reward (i.e., positive) and punishment (i.e., negative) outcomes; *x(t)*: objective outcome;

the ORL separately tracks win frequency to account for win frequency effect:

$EF_{j}\left( t+1 \right)=\left\{ \begin{aligned} EF_{j}\left( t \right)+A_{rew}\cdot\left( sgn（x\left( t \right)）-EF_{j}\left( t \right) \right),x\left( t \right)\geq0 \\ EF_{j}\left( t \right){+A}_{pun}\cdot\left( sgn（x\left( t \right)）-EF_{j}\left( t \right) \right),x\left( t \right)<0 \end{aligned} \right.$

*EF_j_ (t)*: expected outcome frequency; *sgn (x (t))*: returns 1, 0, or -1 for positive, 0, or negative outcome values on trial *t*, respectively.

The ORL model also includes a reversal-learning component for *EF_j_ (t)*.

$$EF_{j'}\left( t+1 \right)=\left\{ \begin{aligned} {EF}_{j'}\left( t \right)+A_{pun}\left( \frac{-sgn (x\left( t \right)}{C}{-EF}_{j'}\left( t \right) \right),x\left( t \right)\geq0 \\ {EF}_{j'}\left( t \right)+A_{rew}\left( \frac{-sgn (x\left( t \right)}{C}{-EF}_{j'}\left( t \right) \right),x\left( t \right)<0 \end{aligned} \right.$$

*EF_j’_ (t)*: the expected outcome frequency of all unchosen decks *j’* on trial *t C*: the number of possible alternative choices for the chosen deck *j*.

The ORL model employs a simple choice perseverance model to capture decision makers’ tendencies to stay or switch decks, irrespective of the outcome:

$PS_{j}\left( t+1 \right)=\left\{ \begin{aligned} \left. \frac{1}{1+K} \right.,D\left( j \right)=j \\ \left. \frac{PS_{j}\left( t \right)}{1+K} \right.,D\left( j \right)\neq j \end{aligned} \right.$

$K=3^{K^{'}}-1$

PS_j_ (t): the perseverance weight of deck *j* on trial *t*; *K*: decay parameter. *K’* ∈ [0,5].

The ORL model integrated value, frequency, and perseverance signals in a linear fashion to generate a single value signal for each deck:

$V_{j}\left( t+1 \right)=EV_{j}\left( t+1 \right){+EF_{j}\left( t+1 \right)\beta}_{F}{+PS_{j}\left( t+1 \right)\beta}_{P}$

β*_P_*，β*_F_* : weights which reflect the effect of outcome frequency and perseverance on total value with respect to the expected value of each deck.

The ORL uses the same softmax function as the VPP to generate choice probabilities. The ORL contains five free parameters (A_rew_, A_pun_, *K*, β*_P_*，β*_F_*).
